# National Health and Modern Problems

**Published:** 1920-07-31

**Authors:** E. Clark-Jones

**Affiliations:** Burbage, Wilts


*To the Editor of* The Hospital.

July 24, 1920.

### National Health and Modern Problems.

*To the Editor of* The Hospital.

Sir,—Looking through an old volume of *Punch* yesterday I came across the following :

*Tourist* (who has been patiently listening to abuse of the rich and plans for betterment of things in general) : "But, my good man, if these changes were carried out it would mean a tremendous social upheaval."

*Old Man* (slapping his thigh) : "Demme, I'd risk it!"

In 1908 that was "good enough for *Punch*." In 1920 it is doubtful if the editor of any journal of humour (real or alleged) would look at it. We have been swept into days of mighty change, of upheavals before which the imagination hitherto would have quailed. And accustomed as we have become by experience to the destruction of order, the seething villainies of anarchy, the beggaring of nations, and the scrapping of empires, we had thought the world, the flesh, and the devil had done their "damnedest." Nothing now could disturb or excite our jaded imaginations. We had, however, not reckoned with your correspondent "Contemplator."

He is distressed because there are unhealthy occupations which men have to follow, which is not remarkable. Further, he has discovered that in these occupations is to be found the root-cause of the admittedly unsatisfactory state of our general national health. But, more remarkable still, he thinks, "it seems reasonable to think that no part of the proposed expenditure (towards the improvement of public health) could be better applied than in careful experiment, in some garden city, to simplify living to approximate more nearly to natural conditions." No one denies that there are callings less healthy than gardening. The trouble arises from the fact that the ingenuity of man's brain outstrips the responsive adaptability of his body. We have to accept it and make the best of it. The human race has burnt its boats. There can be no return to old-world simplicity, and the nation which attempts to cure its physical ills by renouncing the arts of modern industry and by taking to hoeing turnips, will do so more effectually than "Contemplator" would appear to imagine. Not knowing the amount of contemplation "Contemplator" has attempted I do not know whether to advise him to go ahead and do some more of it, or to give it up as a bad job.

But, further, I believe that to saddle the responsibility for the poor standard of health obtaining generally upon the shoulders of such unhealthy industries as do exist is wildly unfair.

The conditions under which the most handicapped of men work to-day are constantly being improved. Hours of labour are curtailed to an ever-increasing extent, and

**The Editor's Letter Box—(*continued*).**

opportunity for relaxation and health-giving exercise are multiplied on every hand.

Under present conditions, the crucial question in respect of the health of the nation at large, *which is far below standard in all sections of the community*, is not "Where do men work?" but "How do they utilise their leisure?"

Yours faithfully,

E. Clark-Jones.

Burbage, Wilts, July 20.

